# Territorial Persistence of Residual *Plasmodium falciparum* Transmission in the Americas: A One Health Perspective

**DOI:** 10.3390/tropicalmed11060151

**Published:** 2026-05-31

**Authors:** Mario J. Olivera

**Affiliations:** Grupo de Parasitología, Instituto Nacional de Salud, Bogotá 111321, Colombia; molivera@ins.gov.co; Tel.: +57-2207700

**Keywords:** malaria, falciparum, disease transmission, spatial analysis, epidemiological monitoring, one health

## Abstract

Malaria reduction in the Americas has slowed despite sustained elimination efforts, suggesting important changes in the spatial organization of residual transmission. This study evaluated whether declining *Plasmodium falciparum* incidence between 2000 and 2023 was associated with increasing geographic concentration, spatial clustering, and territorial persistence across the region, with detailed subnational analysis in Colombia. Temporal trends were assessed using segmented regression, geographic concentration using Gini and Herfindahl–Hirschman indices, and spatial dependence using Moran’s I and local indicators of spatial association. A Municipal Persistence Index (MPI) was developed to identify municipalities characterized by recurrent high transmission over time. Regional incidence declined by 62.7% but showed progressive temporal deceleration, while more than 80% of cumulative burden remained concentrated in four countries. Since 2020, Colombia has contributed nearly 40% of all reported *P. falciparum* cases in the Americas. Within Colombia, high-persistence municipalities largely overlapped with statistically significant spatial clusters concentrated along the Pacific region. These findings suggest that residual *P. falciparum* transmission is increasingly organized into territorially structured persistence systems rather than diffuse endemicity. Advancing malaria elimination may therefore require territorially coordinated One Health strategies aligned with the spatial organization of residual transmission.

## 1. Introduction

Over the past two decades, malaria incidence in the Americas has declined substantially, reflecting expanded access to diagnosis and treatment, vector control implementation, and strengthened surveillance systems [1,2]. These advances have reduced transmission intensity across most endemic countries and narrowed the geographic distribution of disease [3]. As a result, elimination of *Plasmodium falciparum* is considered operationally feasible in much of the region [4]. However, in several endemic settings, progress has slowed despite continued implementation of these interventions, suggesting that remaining challenges are increasingly related to transmission dynamics rather than intervention coverage.

As malaria transmission declines, its epidemiological behavior changes qualitatively. Instead of disappearing uniformly, residual transmission tends to persist within geographically restricted areas where ecological suitability, human mobility, and uneven access to health services converge [5,6]. This shift is consistent with the emergence of territorially structured transmission systems, understood here as spatially connected environments in which ecological conditions, human mobility, and uneven health system access interact to sustain recurrent parasite circulation [7,8].

Evidence from multiple elimination settings indicates that late-stage malaria reduction is characterized by increasing spatial heterogeneity and geographic concentration [2,5]. Early declines typically occur broadly across endemic regions. As transmission declines further, progress slows as it contracts into a limited number of areas. Remaining cases no longer represent diffuse endemicity but rather geographically structured persistence, where repeated local regeneration replaces widespread exposure as the dominant mechanism sustaining transmission [9,10]. When transmission becomes territorially organized, strategies based on uniform expansion of interventions may encounter diminishing returns because the determinants sustaining transmission are unevenly distributed and spatially interconnected [11].

In the Americas, *P. falciparum* increasingly represents this residual component of malaria burden [1]. Transmission persists in forest frontiers, mining-adjacent zones, and border environments shaped by environmental transformation and population mobility [12]. These settings frequently experience recurrent transmission despite declining national incidence, suggesting that persistence operates within connected local systems rather than through isolated outbreaks alone [13]. Determining whether such persistence reflects independent hotspots or spatially coherent structures is essential for elimination planning.

The concept of structural receptivity provides an operational lens for interpreting these dynamics. Rather than referring solely to vector presence, receptivity can be understood more broadly as the territorial convergence of ecological suitability, climatic permissiveness, human connectivity, and health system reach. This convergence enables transmission to recur in the same locations over time [14,15]. When these components overlap spatially, transmission may remain stable at low levels even when biomedical tools are widely available. Under these conditions, elimination difficulty arises not from the absence of interventions but from their application at scales that do not match the spatial organization of residual transmission.

Within this context, the One Health perspective offers a framework to translate epidemiological structure into coordinated action [16]. By integrating environmental conditions, human mobility, and health system performance, this approach enables identification of territorially connected transmission systems as operational units for intervention [17]. Rather than treating residual malaria as independent local events, elimination strategies can focus on persistent transmission corridors where interacting determinants sustain transmission. Importantly, this study adopts a structurally informed One Health perspective, in which biological processes are understood as dynamically interacting with social, economic, and institutional factors that shape transmission persistence across territories.

This study evaluates whether the slowing reduction in *P. falciparum* transmission in the Americas is associated with increasing geographic concentration, spatial clustering, and territorial persistence. Using regional surveillance data and detailed subnational analysis in Colombia, temporal deceleration, geographic concentration, and spatial clustering are assessed across scales. These patterns are interpreted within a territorially and structurally informed One Health framework to examine how residual transmission becomes increasingly organized within persistent territorial systems in late-stage elimination settings.

## 2. Materials and Methods

### 2.1. Study Design

A multiscale ecological design was used to evaluate spatial and temporal patterns of *P. falciparum* transmission in the Americas from 2000 to 2023, including detailed subnational analysis in Colombia. Analyses were conducted using aggregated country- and municipality-level surveillance data, and no individual-level information was used.

The analysis was descriptive and exploratory, focusing on temporal dynamics, geographic concentration of burden and spatial dependence patterns. Findings were interpreted within a territorial epidemiological systems framework, and no causal inference was attempted.

### 2.2. Data Sources

Annual country-level *P. falciparum* case counts (2000–2023) were obtained from the Pan American Health Organization regional malaria surveillance database and official national surveillance reports. Species-disaggregated records were used to isolate falciparum transmission from total malaria burden.

Subnational data were obtained from the Colombian national public health surveillance system (SIVIGILA). Colombia was selected for detailed subnational analysis because it consistently contributed a substantial share of regional falciparum burden and encompassed diverse ecological and transmission settings representative of patterns observed across the Americas. Municipalities were included if they reported confirmed malaria transmission during at least two consecutive years within the study period. This criterion was used to identify municipalities with evidence of recurrent transmission while reducing the influence of isolated or potentially imported events.

Annual municipal population denominators were obtained from the National Administrative Department of Statistics (DANE). Annual Falciparum Incidence (AFI) was calculated as the number of confirmed *P. falciparum* cases per 1000 population at risk. The population at risk was defined using rural and dispersed population estimates in endemic municipalities, consistent with regional surveillance standards.

Country-level species proportions were calculated to describe the relative contribution of *P. falciparum* to total malaria burden. Countries were descriptively categorized as falciparum-dominant, mixed transmission or vivax-dominant.

Contextual environmental information describing forest disturbance, hydrological conditions and mining activity was compiled from national monitoring reports. Data on the presence of *Anopheles* vectors were obtained from entomological surveillance summaries of the Colombian National Institute of Health and biodiversity occurrence databases. These sources were used for contextual interpretation of spatial patterns rather than as predictors in statistical models.

### 2.3. Temporal Trend Analysis

Long-term regional trends in *P. falciparum* cases were evaluated using log-linear regression:

$$log(Y_{t})=\beta_{0}+\beta_{1}t.$$

where $Y_{t}$ represents annual regional *P. falciparum* cases.

To describe changes in the velocity of decline, segmented regression was applied across predefined epidemiological intervals (2000–2010, 2010–2016, 2016–2019, and 2019–2023). These intervals defined a priori to represent epidemiologically interpretable phases of regional decline rather than statistically estimated breakpoints.

Annual percent change (APC) for each interval to quantify variation in the rate of decline:

$$APC=(e^{\beta }-1)\times 100.$$

To evaluate whether temporal decline was better represented by segmented dynamics rather than a constant linear reduction, segmented and unsegmented log-linear models were compared using Akaike Information Criterion (AIC), Bayesian Information Criterion (BIC), adjusted $R^{2}$, and residual variance estimates. This comparison was intended as an exploratory assessment of temporal nonlinearity rather than formal breakpoint testing.

Residual diagnostics were visually inspected to assess departures from linearity during the late elimination phase.

### 2.4. Geographic Concentration

Geographic concentration of *P. falciparum* burden was quantified using inequality metrics. The Gini coefficient was used to assess distributional inequality across spatial units:

$$G=1-\sum \left(X_{k}-X_{k-1}\right)\left(Y_{k}+Y_{k-1}\right).$$

And the Herfindahl–Hirschman Index (HHI) was used to measure concentration within a subset of countries or municipalities:

$$HHI=\sum s_{i}^{2}.$$

where $s_{i}$ represents the proportional contribution of each country or municipality to total cases. Although commonly used in economic analyses, the HHI can be applied more generally as a concentration metric to describe how total burden is distributed across spatial units. Values were interpreted comparatively across time and scale rather than against fixed categorical thresholds.

Pareto-type distributions were constructed to identify the proportion of spatial units accounting for the majority of cases. Municipal concentration was additionally summarized by estimating the contribution of the highest-burden municipalities to total subnational cases.

### 2.5. Spatial Dependence Analysis

Spatial dependence of municipal AFI in Colombia was evaluated using Global Moran’s I with first-order queen contiguity spatial weights:

$$I=\frac{n}{W}\frac{\sum_{i}\sum_{j}w_{ij}(x_{i}-\bar{x})(x_{j}-\bar{x})}{\sum_{i}(x_{i}-\bar{x}{)}^{2}}.$$

Local Indicators of Spatial Association (LISA) were used to identify statistically significant high–high and low–low clusters.

To approximate stable recent spatial structure, AFI values were averaged across the most recent five-year period (2019–2023). This aggregation reduced stochastic interannual variability in low-incidence settings and emphasized persistent spatial patterns.

Sensitivity analyses using alternative temporal aggregations, including three-year averages and annual estimates, yielded qualitatively similar clustering patterns, although with greater variability in low-incidence municipalities.

As a complementary assessment of spatial consistency, exploratory spatial scan statistics were performed using SaTScan with a discrete Poisson model. Municipal case counts aggregated for 2019–2023 were analyzed using population-at-risk denominators to identify high-incidence spatial clusters. The maximum spatial cluster size was restricted to 15% of the population at risk to prioritize localized persistent transmission structures.

These analyses were intended as exploratory validation of the spatial dependence patterns identified through Moran’s I and LISA. Statistical significance was assessed using 999 permutations, and multiple testing was addressed through false discovery rate correction.

### 2.6. Municipal Persistence Analysis

To characterize long-term territorial persistence of transmission, a Municipal Persistence Index (MPI) was calculated as the proportion of study years during which municipal AFI remained above the 75th percentile of the national AFI distribution.

$$MPI_{i}=\frac{{\mathrm{Number}\;\mathrm{of}\;\mathrm{years}\;\mathrm{with}\;\mathrm{AFI}}_{i}>P75}{\mathrm{Total}\;\mathrm{study}\;\mathrm{years}}.$$

where $MPI_{i}$ represents the persistence index for municipality i, AFI corresponds to the Annual *P. falciparum* Incidence, and P75 represents the national 75th percentile threshold of annual AFI values.

The MPI was used descriptively to identify municipalities characterized by recurrent high transmission over time rather than isolated epidemic events. Municipalities were classified into low persistence (<25% of years above threshold), moderate persistence (25–50%), and high persistence (>50%).

To evaluate convergence between complementary spatial approaches, overlap was assessed between municipalities with high persistence, statistically significant LISA high–high clusters, and exploratory SaTScan-defined clusters. The overlap analysis was used as a spatial triangulation strategy to evaluate convergence between temporal persistence, local spatial autocorrelation, and statistically significant clustering.

### 2.7. Structural Interpretation Framework

For analytical consistency, transmission corridors were operationally defined as spatially contiguous municipal clusters exhibiting persistent high transmission over time. A corridor was defined as a contiguous set of at least three adjacent municipalities exhibiting either (i) high incidence in at least three of the most recent five years or (ii) a multiyear mean AFI above the 75th percentile of the national distribution.

The requirement of at least three adjacent municipalities was used as a conservative operational criterion to distinguish territorially coherent spatial structures from isolated hotspots or simple pairwise adjacency. This definition was heuristic and intended to support the identification of connected persistence systems rather than to establish a universal epidemiological threshold.

The concept was used descriptively to characterize territorially coherent persistence systems rather than to infer directly measured mobility or parasite flow networks. Spatial analyses were conducted using GeoDa and the spdep package in R version 4.3.2 (R Foundation for Statistical Computing, Vienna, Austria).

### 2.8. Ethical Considerations

The study used anonymized, aggregated public surveillance data and did not involve identifiable personal information. In accordance with Colombian Resolution No. 8430 of 1993 and international guidelines for secondary analysis of public health data, the study was considered minimal risk and did not require formal ethical committee approval.

## 3. Results

### 3.1. Long-Term Regional Decline and Temporal Deceleration

**Figure 1:**
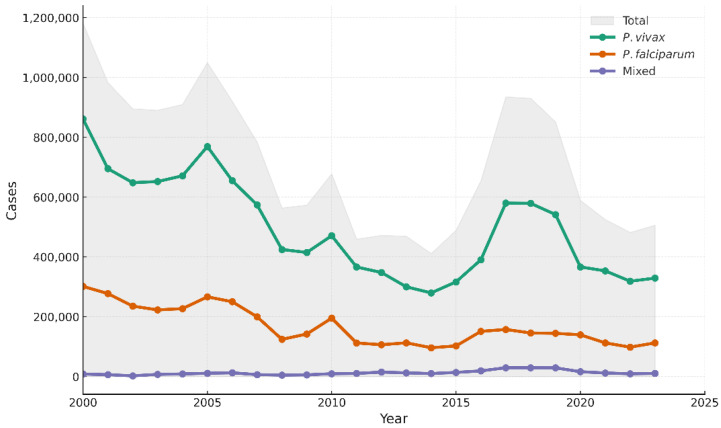
Temporal trends of malaria cases in the Americas, 2000–2023. Annual reported cases by species. The shaded area represents total malaria burden and lines indicate *P. vivax*, *P. falciparum*, and mixed infections. The sustained decline followed by inter-annual variability illustrates temporal deceleration compatible with an epidemiological plateau.

Between 2000 and 2023, reported *P. falciparum* cases in the Americas declined from 301,390 to 112,455, corresponding to a 62.7% reduction (Figure 1). Declines were observed across most endemic countries, although magnitude and timing varied between settings.

Despite this overall reduction, the rate of decline was not constant over time. Segmented analysis showed a progressive deceleration in the velocity of reduction. During 2000–2010, annual decreases were approximately 4–5%; between 2010 and 2016, the rate declined to ~3% per year; and during 2016–2019, reductions slowed further to ~1–2% annually, accompanied by increased interannual variability. From 2019 to 2023, a moderate decline resumed but remained below early-period levels.

Comparison between segmented and unsegmented log-linear models indicated improved fit for the segmented specification, indicating that malaria decline did not follow a constant linear trajectory across the study period (Table 1).

The segmented model showed lower AIC and BIC values, higher adjusted $R^{2}$ and reduced residual variance compared with the unsegmented model. Residual inspection of the unsegmented model suggested increasing departures from linearity during the late elimination phase, consistent with temporal stabilization of transmission at lower incidence levels.

Overall, the time series indicates a transition from rapid early reductions to a phase of temporal deceleration, with fluctuations consistent with stabilization of transmission at lower levels rather than continued linear decline. Additional descriptive temporal, concentration, and spatial metrics are presented in Supplementary Table S1.

### 3.2. Geographic Concentration of Regional Falciparum Burden

**Figure 2:**
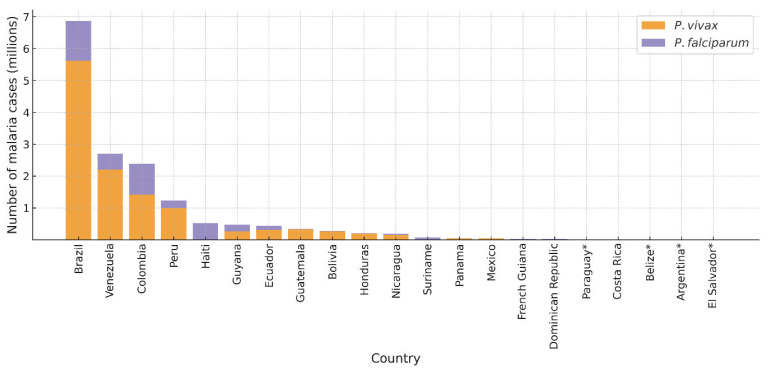
Cumulative malaria cases by country in the Americas, 2000–2023. Stacked bars represent total malaria burden by species, with contributions from *P. vivax* and *P. falciparum*. A limited number of countries account for the majority of regional burden, indicating strong geographic concentration of transmission. Countries marked with an asterisk (*) have achieved malaria elimination.

Declining incidence was accompanied by increasing geographic concentration of *P. falciparum* transmission. While total malaria cases (including *P. vivax*) were more widely distributed across countries (Figure 2), *P. falciparum* burden was highly concentrated.

**Figure 3:**
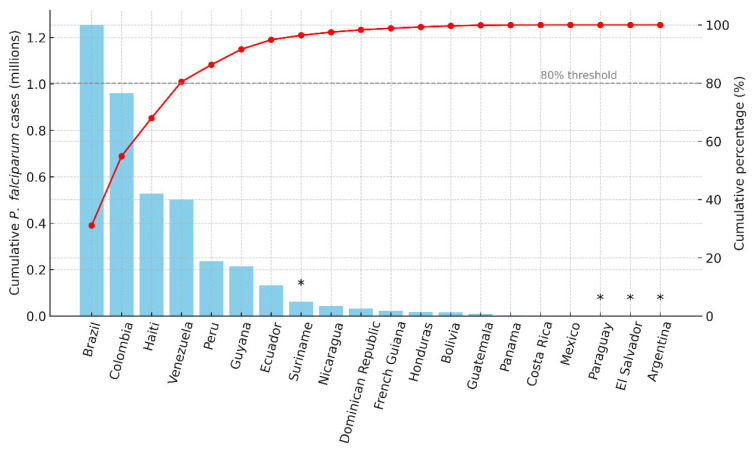
Pareto distribution of regional *P. falciparum* burden. Countries ranked by cumulative contribution. The 80% threshold is reached by fewer than half of endemic countries, demonstrating persistent concentration across the study period. (*) Malaria elimination achieved.

Between 2000 and 2023, four countries, Brazil (31%), Colombia (24%), Haiti (13%), and Venezuela (12%), accounted for over 80% of regional *P. falciparum* cases (Figure 3). This concentration remained stable over time. During 2019–2023, the same countries accounted for approximately 85% of reported infections, with limited variation in country rankings.

Inequality metrics confirmed this pattern. The Gini coefficient increased from approximately 0.68 to 0.75, and HHI values ranged between 0.18 and 0.23, indicating progressive concentration of burden within a limited subset of countries. These findings indicate that regional decline occurred alongside consolidation of transmission within a relatively stable group of high-contributing countries rather than geographic redistribution across the region.

### 3.3. Species Composition over Time

Regional reductions in incidence occurred alongside persistent heterogeneity in species composition *P. falciparum* represented approximately one-quarter of malaria cases at the beginning of the study period and remained above one-fifth by 2023 (Figure 1), indicating continued epidemiological relevance.

**Figure 4:**
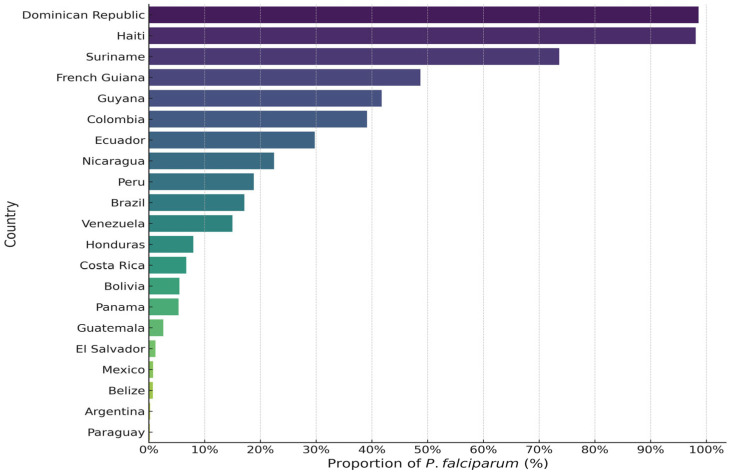
Country-level proportion of *P. falciparum* among total malaria cases, 2000–2023. The figure distinguishes epidemiological profiles ranging from falciparum-dominant systems to mixed-species transmission settings.

However, countries differed substantially in species structure (Figure 4). Some settings, including Haiti and the Dominican Republic, exhibited near-exclusive *P. falciparum* transmission, while others such as Suriname and French Guiana showed high falciparum predominance despite relatively low absolute burden.

In contrast, countries with large total malaria burden, including Brazil, Peru, and Nicaragua, maintained mixed-species transmission with lower *P. falciparum* proportions. Colombia and Venezuela displayed intermediate profiles.

These patterns indicate that regional malaria epidemiology reflects two distinct but related dimensions: concentration of absolute burden in a limited number of countries and variation in species dominance within countries, reflecting distinct transmission environments.

### 3.4. Subnational Spatial Organization of Transmission in Colombia

At the subnational level, *P. falciparum* transmission in Colombia showed marked spatial heterogeneity. Although cumulative cases exceeded 370,000 during the study period, distribution was highly uneven: the five municipalities with the highest burden accounted for more than one-third of cases, and the ten highest accounted for more than half (Gini ≈ 0.61).

Several of these high-burden municipalities have historically contributed disproportionately to malaria transmission, suggesting that the observed spatial concentration reflects persistent transmission systems rather than newly emerging hotspots.

Absolute burden and transmission intensity were not fully aligned. Municipalities with the highest cumulative case counts did not necessarily correspond to those with the highest mean AFI, indicating persistent transmission intensity in smaller populations rather than population-size effects alone.

Species composition differed across transmission environments. Several municipalities in the Pacific region exhibited both high incidence and a high proportion of *P. falciparum*, whereas other areas maintained mixed-species profiles.

**Figure 5:**
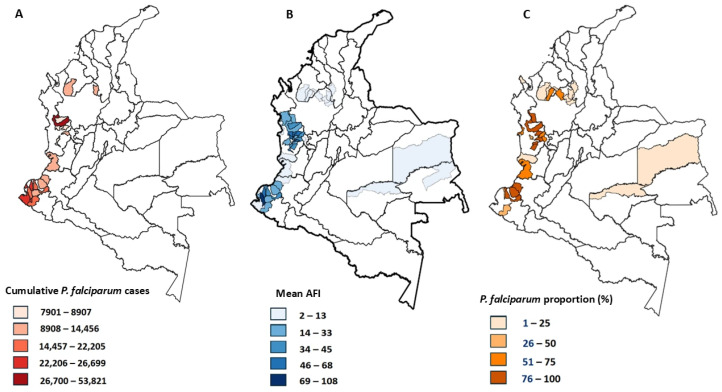
Municipal-level indicators of *P. falciparum* transmission in Colombia during the study period. (**A**) Cumulative falciparum cases by municipality. (**B**) Mean AFI per 1000 population at risk. (**C**) Proportion of *P. falciparum* among total malaria cases. The three indicators reveal complementary dimensions of transmission: concentration of absolute burden, persistence of transmission intensity, and species predominance. High-burden municipalities are geographically clustered in the Pacific region, where several territories simultaneously show elevated incidence and high *P. falciparum* proportion, indicating territorially stable transmission environments rather than isolated outbreaks.

Together, these indicators (burden concentration, incidence intensity, and species composition) co-occurred geographically in specific regions, defining localized transmission environments rather than broadly distributed endemicity (Figure 5).

### 3.5. Spatial Clustering of Transmission

Spatial dependence analysis demonstrated statistically significant clustering of *P. falciparum* transmission. Global Moran’s I for mean AFI (2019–2023) was approximately 0.34 (*p* < 0.001), indicating non-random spatial structure. These clustering patterns reflect the spatial organization of transmission during the late phase of decline, rather than long-term average conditions.

LISA identified contiguous high–high clusters primarily in the Pacific region, including municipalities in Choco, Nariño, Cauca, and southern Antioquia (Figure 5). Low–low clusters were observed outside these regions and remained spatially separated from high-incidence areas.

High-incidence municipalities formed continuous geographic groupings rather than isolated hotspots. This configuration is consistent with territorially coherent clustering across adjacent municipalities, indicating spatially structured persistence rather than independent local events.

### 3.6. Territorial Persistence of Transmission Systems

Persistence analysis indicated that residual transmission was not only spatially clustered but also temporally recurrent within specific municipalities.

**Figure 6:**
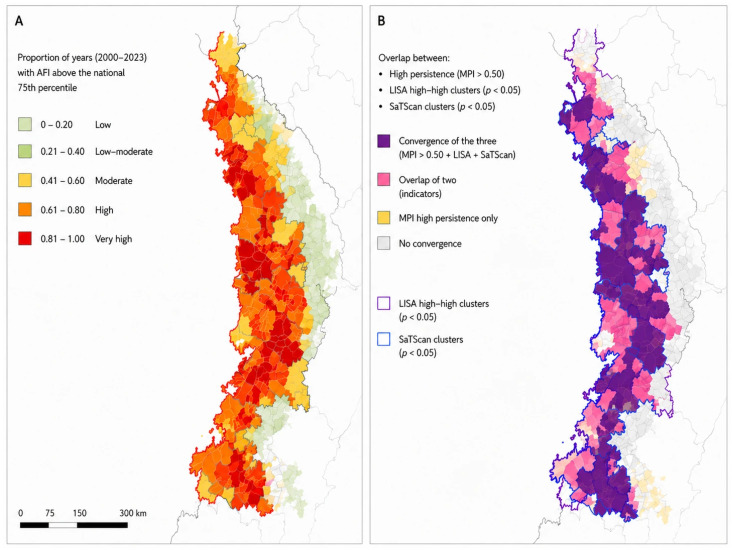
Territorial persistence and spatial convergence of residual *P. falciparum* transmission in Colombia (2000–2023). (**A**) displays the Municipal Persistence Index, representing the proportion of study years during which municipal AFI remained above the national 75th percentile. (**B**) illustrates the spatial convergence between high persistence municipalities, LISA high–high clusters, and exploratory SaTScan clusters. Persistent transmission systems were concentrated primarily within the Pacific region, including municipalities in Choco, Nariño, Cauca, and southern Antioquia.

Municipalities located within the Pacific region exhibited the highest MPI values, with several municipalities remaining above the national AFI 75th percentile during more than 50% of study years (Figure 6). High-persistence municipalities largely overlapped with statistically significant LISA high–high clusters and exploratory SaTScan clusters, particularly in municipalities spanning Choco, Nariño, Cauca, and southern Antioquia.

In contrast, municipalities outside the principal persistence systems generally exhibited lower MPI values and more intermittent transmission dynamics.

The spatial convergence between persistence, local autocorrelation, and scan-based clustering identified geographically coherent areas of recurrent transmission concentrated primarily within the Pacific region of Colombia (Table 2).

### 3.7. Multiscale Spatial Organization of Residual Transmission

Across spatial scales, declining incidence coexisted with increasing concentration and spatial aggregation. At the regional level, *P. falciparum* burden remained concentrated within a limited number of countries, while at the subnational level in Colombia transmission was organized into contiguous municipal clusters.

These clusters were located in areas with local connectivity, including riverine and road-linked settlements in forest frontier and mining-associated environments. Repeated case reporting across neighboring municipalities over multiple years was observed, indicating recurrent spatial configurations.

**Figure 7:**
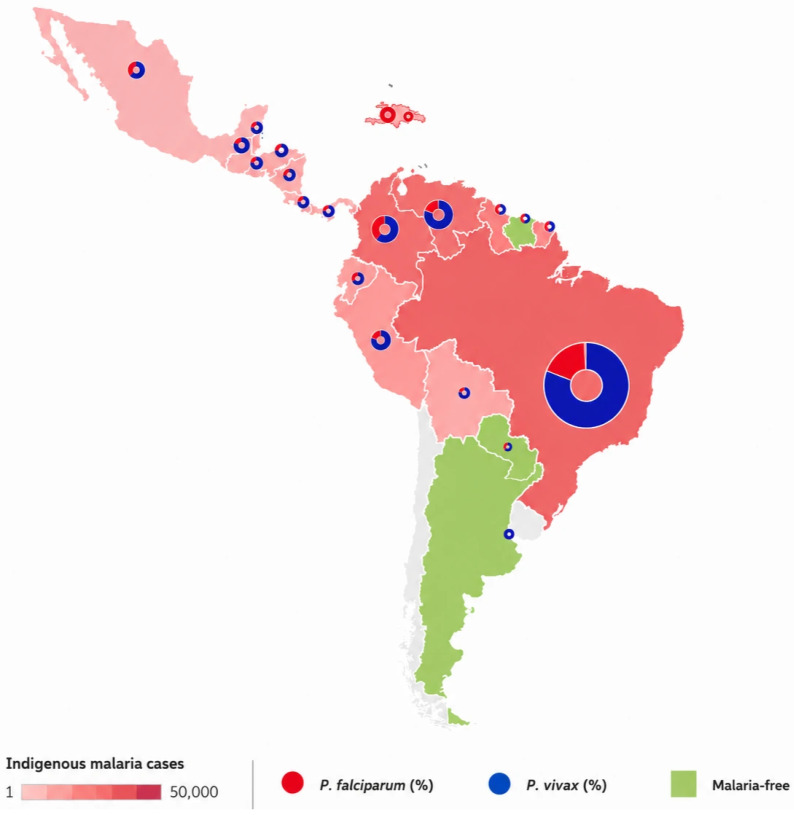
Spatial distribution of *P. falciparum* burden across the Americas. Circle size represents absolute falciparum burden, and color proportion indicates the relative contribution of *P. falciparum* compared with other malaria species. High-burden areas form geographically contiguous zones rather than isolated locations, illustrating transnational transmission concentration across northern South America and Hispaniola.

The convergence of geographic concentration at the continental scale and clustering at the municipal scale indicates that residual transmission operates within territorially structured persistence systems rather than isolated hotspots (Figure 7).

Three consistent patterns emerged across analyses: (1) temporal deceleration in the rate of decline, (2) geographic concentration of burden within a limited number of countries, and (3) spatial clustering into contiguous high-incidence municipal groupings. These patterns co-occurred across scales and are consistent with increasing territorial concentration of residual transmission during the late elimination phase.

## 4. Discussion

Declining *P. falciparum* transmission in the Americas does not reflect a uniform trajectory toward elimination. Instead, it reveals a spatial reorganization in which residual transmission becomes increasingly concentrated within territorially structured persistence systems. These systems are not newly formed but reflect persistent spatial structures that become more evident as overall transmission declines. Across scales, persistence is maintained within contiguous areas shaped by overlapping ecological conditions, human mobility, and health system dynamics. This configuration suggests that transmission is sustained through spatially structured persistence rather than isolated hotspots. A structurally informed One Health perspective provides a framework to interpret these patterns as the product of interacting ecological, social, and institutional processes operating across geographically connected territories.

Temporal patterns provide an initial indication of this shift. Early reductions were relatively rapid and widespread, whereas subsequent declines slowed despite continued intervention coverage [18]. This nonlinear trajectory is consistent with observations from other pre-elimination settings, where transmission becomes increasingly dependent on localized ecological and social conditions rather than generalized exposure [19,20]. Under these conditions, further reductions become progressively more difficult because residual transmission is maintained within environments that repeatedly support parasite circulation. As incidence declines, transmission contracts into geographically connected systems in which local regeneration replaces widespread exposure.

Geographic concentration reinforces this interpretation. A limited number of countries consistently accounted for the majority of falciparum cases across all time periods, and this distribution remained stable despite temporal fluctuations [21,22,23]. Rather than shifting geographically, transmission appears to stabilize within historically receptive environments [24,25]. Importantly, concentration of absolute burden and variation in species composition represent distinct epidemiological dimensions: some countries contribute a large share of cases, while others maintain high *P. falciparum* predominance despite lower total burden [26]. This divergence suggests that persistence reflects stable transmission environments rather than differences in population size or reporting volume. Although part of this concentration may reflect population size and ecological suitability, the increasing geographic concentration and spatial dependence observed indicate that transmission is becoming progressively structured rather than uniformly distributed.

At the subnational level, the same configuration emerges at finer spatial scales. In Colombia, a small proportion of municipalities consistently contributed a large share of cases and formed contiguous clusters of elevated incidence [12,27]. The observed spatial dependence indicates that transmission is not randomly distributed but concentrated within territorially coherent municipal groupings [13,28]. These configurations are consistent with the operational definition of transmission corridors used in this study, understood as spatially contiguous persistence structures rather than directly measured mobility networks [29,30]. Together, these findings suggest that residual transmission persists within geographically coherent systems characterized by recurrent transmission over time.

The MPI did not simply identify municipalities with elevated incidence, but territories characterized by repeated temporal recurrence of transmission over more than two decades. The spatial overlap between persistence, local autocorrelation, and scan-based clusters suggests that residual *P. falciparum* transmission in Colombia is increasingly organized into territorially structured persistence systems rather than diffuse endemicity.

From a structural One Health perspective, these systems likely reflect interactions among ecological suitability, mobility dynamics, extractive economies, environmental transformation, institutional vulnerability, and uneven healthcare access operating across geographically connected territories. Many of the identified persistence systems coincide with areas historically shaped by mining activity, forest frontier expansion, riverine mobility, and limited health system reach.

Under this interpretation, malaria persistence reflects not only biological receptivity, but also socioecological organization structured through territorial inequalities and recurrent human–environment interactions. Rather than viewing residual malaria exclusively as an entomological or clinical problem, these findings support a broader interpretation in which transmission persistence emerges from interacting ecological and social conditions operating across territories.

Taken together, these findings suggest that the slowing of malaria elimination in the Americas reflects increasing territorial concentration of residual transmission rather than continued homogeneous decline [31]. Under this configuration, transmission persists within recurrent socioecological systems that may be less responsive to uniformly implemented interventions [17].

The analyses are descriptive and do not establish causal relationships. Environmental characteristics, extractive activities, and connectivity patterns were used to interpret spatial coherence, and their role should be understood as contextual and associative rather than causal [28]. The persistence of clustered transmission is therefore interpreted as compatible with structurally receptive environments rather than attributed to specific determinants [26,32].

Within this context, structural receptivity provides a useful interpretive framework. In this study, receptivity reflects the territorial convergence of ecological suitability, climatic permissiveness, human mobility, and access to diagnosis and treatment [8,15].

These findings have direct implications for malaria elimination strategies. When transmission is territorially structured, approaches based on uniform expansion of interventions may produce diminishing returns [6]. Local reductions can be offset by continued transmission in neighboring areas, particularly where short-distance mobility and shared ecological conditions facilitate parasite circulation [7]. In such settings, isolated interventions applied at administrative levels may be insufficient to interrupt transmission if adjacent areas remain receptive [33]. Within a structurally informed One Health framework, intervention strategies should address environmental processes, human mobility, economic activity, and health system reach operating across connected territories [34].

A territorially oriented application of this framework emphasizes the identification of operational units defined by spatially connected transmission systems rather than administrative boundaries [6]. In this approach, interventions are organized across contiguous municipalities where transmission is maintained, integrating vector control, case management, and environmental management at the scale at which persistence occurs [31]. This perspective shifts the focus from isolated local control toward integrated strategies that reflect the spatial organization of transmission [34].

In practice, territorially coordinated strategies may include synchronized case detection, targeted vector control, and improved access to diagnosis and treatment across contiguous municipalities where recurrent transmission persists [29].

The findings also highlight the importance of distinguishing between spatial correlation and functional connectivity. While this study identifies geographically contiguous clusters compatible with transmission corridors, it does not directly model human mobility or parasite flow between locations [7,35]. Therefore, the concept of transmission corridor should be interpreted as a spatially coherent pattern of persistence consistent with local connectivity, rather than as a directly measured network of transmission pathways [6].

Although Colombia was selected as a detailed subnational case due to data availability and epidemiological relevance, consistent high-resolution subnational data were not uniformly available across all countries in the region. Therefore, detailed spatial analyses were restricted to Colombia as a representative case study. Caution is needed in generalizing specific parameter values, such as cluster size or Moran’s I magnitude, to other countries [26,31]. However, the broader pattern of transmission contraction into spatially concentrated and territorially connected persistence systems is likely to be relevant across multiple endemic contexts, even if its specific expression differs by setting [36].

This study has several limitations. Analyses relied on aggregated surveillance data that may be affected by underreporting and variation in diagnostic coverage across time and locations. Spatial analyses were based on administrative boundaries rather than direct mobility networks. Surveillance heterogeneity may have influenced the apparent spatial distribution of transmission, particularly in remote municipalities with limited access to diagnostic services. Environmental and institutional variables were used for contextual interpretation rather than causal inference. Additionally, comparable high-resolution subnational data were not consistently available across all endemic countries, limiting the possibility of equivalent fine-scale analyses beyond Colombia. Despite these limitations, the persistence of clustering across multiple analytical approaches and over extended temporal periods suggests that the observed patterns are unlikely to be explained solely by reporting variability.

Together, these findings suggest that residual *P. falciparum* transmission in the Americas is increasingly concentrated within territorially structured persistence systems rather than diffuse endemicity. Late-stage elimination therefore appears constrained not only by transmission intensity, but also by the spatial mismatch between residual transmission dynamics and the scale at which interventions are implemented. From a structurally informed One Health perspective, advancing elimination may require territorially coordinated strategies adapted to persistent transmission systems.

## 5. Conclusions

Declining *P. falciparum* transmission in the Americas is increasingly characterized by geographic concentration, spatial clustering, and long-term territorial persistence rather than uniform regional decline. Residual transmission appears concentrated within territorially structured socioecological systems where ecological suitability, mobility dynamics, and uneven health system reach may interact to sustain recurrent transmission.

The convergence of temporal deceleration, geographic concentration, persistence analysis, and spatial clustering suggests that late-stage malaria elimination is constrained not only by transmission intensity, but also by the spatial organization of residual transmission. Under these conditions, interventions implemented uniformly across administrative units may become progressively less effective.

From a structurally informed One Health perspective, advancing malaria elimination may therefore require territorially coordinated strategies aligned with the spatial structure of persistent transmission systems. Identifying and targeting geographically coherent areas of recurrent transmission could improve the effectiveness of elimination efforts in settings where malaria increasingly persists despite broader regional decline.

## Figures and Tables

**Table 1:** Comparison of temporal trend models for regional *P. falciparum* transmission in the Americas, 2000–2023.

Model	Parameters	Adjusted $R^{2}$	Residual Variance	AIC	BIC
Unsegmented log-linear	2	0.71	0.084	412.6	418.9
Segmented log-linear	5	0.86	0.041	389.3	401.7

**Table 2:** Spatial clustering robustness and persistence metrics in Colombian municipalities.

Metric	Result	Interpretation
Global Moran’s I	0.34 (p<0.001)	Significant spatial clustering
Primary SaTScan cluster RR	3.8	Elevated localized transmission
Secondary SaTScan cluster RR	2.1	Moderate persistent aggregation
Municipalities with high persistence (>50%)	18	Stable recurrent transmission
Overlap between LISA and SaTScan clusters	81%	Substantial spatial convergence
Mean persistence index within Pacific persistence system	0.64	Long-term territorial persistence

## Data Availability

The data that supports the findings of this study are available from the corresponding author upon reasonable request via email.

## Supplementary Materials

The following supporting information can be downloaded at: https://www.mdpi.com/article/10.3390/tropicalmed11060151/s1, Table S1: Multiscale metrics of temporal, geographic, and spatial structure of *P. falciparum* transmission in the Americas and Colombia.

## Abbreviations

The following abbreviations are used in this manuscript:

AFI	Annual Falciparum Incidence
LISA	Local Indicators of Spatial Association
APC	Annual percent change
