# Supplementary material for: Territorial Persistence of Residual Plasmodium falciparum Transmission in the Americas: A One Health Perspective
Source: Trop Med Infect Dis. 2026 May 31;11(6):151. doi: 10.3390/tropicalmed11060151 (PMC13308429; doi:10.3390/tropicalmed11060151)
Supplement: Supplementary file 1 [file tropicalmed-11-00151-s001.zip › tropicalmed-4192951-supplementary.pdf]

Table S1. Multiscale metrics of temporal, geographic, and spatial structure of *Plasmodium falciparum* transmission in the Americas and Colombia

Scale	Metric	Time period	Value (approx.)	Interpretation
**Regional (Americas)**	Total cases	2000	301,390	Baseline burden
	Total cases	2023	112,455	62.7% reduction
	Annual percent change (APC)	2000–2010	−4.5%	Rapid decline phase
	APC	2010–2016	−3.0%	Moderate decline
	APC	2016–2019	−1.5%	Marked deceleration
	APC	2019–2023	−2.0%	Partial recovery of decline
	Gini coefficient	2000–2005	0.68	Moderate concentration
	Gini coefficient	2019–2023	0.75	High concentration
	HHI	2000–2005	0.18	Moderate concentration
	HHI	2019–2023	0.23	Increased concentration
	Top 4 countries contribution	2000–2023	>80%	Strong geographic concentration
**Subnational (Colombia)**	Total cases	2000–2023	~370,000	High cumulative burden
	Gini coefficient (municipal)	2000–2023	0.61	Uneven distribution
	Top 5 municipalities	Contribution	>33%	High concentration
	Top 10 municipalities	Contribution	>50%	Strong concentration
	Mean AFI (selected clusters)	2019–2023	>10 per 1,000	Persistent transmission intensity
	Global Moran's I	2019–2023	0.34 ($p < 0.001$)	Significant spatial clustering

Scale	Metric	Time period	Value (approx.)	Interpretation
	LISA clusters	2019–2023	High–high (Pacific region)	Contiguous clustering
	Corridor definition threshold	—	≥3 municipalities	Operational spatial unit
